# Onychomycosis caused by *Chaetomium globosum*: an uncommon fungal nail infection

**DOI:** 10.22034/cmm.2025.345421.1641

**Published:** 2025-02-01

**Authors:** Astha Yadav, Prashant Gupta, Gopa Banerjee, Raj Kumar Kalyan, Mohd Saqib Hasan

**Affiliations:** 1 Senior Resident, King George’s Medical College, Lucknow, India; 2 Professor, King George’s Medical College, Lucknow, India

**Keywords:** *Chaetomium globosum*, Nail infection, Non-dermatophyte mold, Onychomycosis

## Abstract

**Background and Purpose:**

Onychomycosis is predominantly caused by dermatophytes; however, non-dermatophytic molds are emerging as opportunistic pathogens. These infections pose diagnostic and therapeutic challenges due to their rarity, variable drug susceptibility, and frequent misidentification.

**Case report:**

This study reported a case of onychomycosis in a 46-year-old immunocompetent male caused by *Chaetomium globosum*. Diagnosis was established by direct potassium hydroxide microscopy of nail clippings, which revealed septate hyphae, and fungal culture that produced pigmented colonies with characteristic microscopic features. Antifungal susceptibility testing, performed according to CLSI M38 guidelines, showed sensitivity to itraconazole (MIC=0.25 µg/mL). The patient was treated with oral itraconazole and topical 5% nail lacquer (Nailrox, India), resulting in significant clinical improvement.

**Conclusion:**

This case highlighted the importance of accurate fungal identification, which is crucial in atypical nail infections, and occupational exposure should be considered in non-dermatophytic onychomycosis.

## Introduction

*Chaetomium globosum* is a rare causative agent of onychomycosis, typically found in soil, decaying plant material, and other organic matter. The genus *Chaetomium* is classified under the family Chaetomiaceae, class Sordariomycetes, and phylum Ascomycota [ 1 ]. It is a dematiaceous non-dermatophyte fungus, which is commonly found in the environment, but its involvement in human infections, particularly nail infections, is infrequent and often underreported. Its identification requires careful mycological workup, including direct microscopy and culture on appropriate media, followed by microscopic identification of characteristic perithecia and ascospores [ 2 ].

This case study aimed to report a case of onychomycosis in a patient who presented with brownish-yellow to black nail discoloration and subungual hyperkeratosis involving the right-hand nail. Direct microscopic examination of nail scrapings using potassium hydroxide preparation revealed septate hyphae. Cultures on Sabouraud's dextrose agar without cycloheximide consistently produced fast-growing colonies, initially appearing velvety white before turning dark grey to brown. Slide culture analysis revealed brown-colored septate hyphae, perithecia, and ascospores, confirming the presence of *Chaetomium globosum*. Species-level identification was performed by MALDI-TOF (Matrix-assisted laser desorption ionization–time of flight) mass spectrometry. This report contributed to increasing awareness about rare fungal etiologies in nail infections, particularly in patients exposed to soil and plant material.

## Case Presentation

A 46-year-old male visited our department with progressive nail fragility and breakage of the right-hand nails over the past year. He presented with progressive nail discoloration. The discoloration began as a faint yellowish spot and gradually spread to involve the entire nail. He did not recall any trauma or any injury to nails or any other history of long-term administration of steroids or drugs. The patient had no known chronic illnesses, including diabetes, cardiovascular disease, or immunosuppressive conditions. He was a mason by occupation and a resident of West Champaran, Bihar, India.

**Figure 1:**
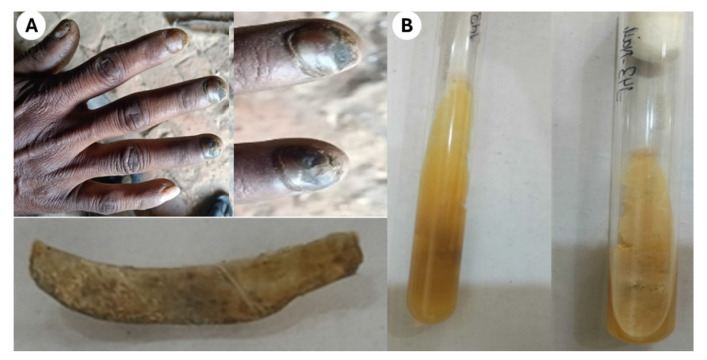
**(A)** Yellow brownish to black discoloration with hyperkeratosis on the right hand nails. Close-up view of the right-hand nails and Nail clipping, **(B)** Obverse and reverse surface of Sabouraud’s dextrose agar plate after incubation at 25 ᵒC for 5 days

On examination, the nail appeared thickened, brittle, and discolored with a yellowish-brown to black hue. The nail plate was dystrophic, with irregular ridging and subungual hyperkeratosis. There was no evidence of periungual inflammation or involvement of skin, including the scalp. General physical examination results were unremarkable (Figure 1A).

Nail clippings were collected using a sterile nail cutter and subjected to 40% potassium hydroxide digestion. Direct microscopy revealed septate, branched hyphae.

Cultures on Sabouraud’s Dextrose Agar with chloramphenicol incubated at 25 °C showed rapid growth of a cottony white mycelial colony, which later turned grayish-green with dark speckles and a brown reverse. Lactophenol cotton blue mount revealed large, lemon-shaped perithecia with terminal setae, which is the characteristic of *C. globosum* (Figure 1B). 

**Figure 2:**
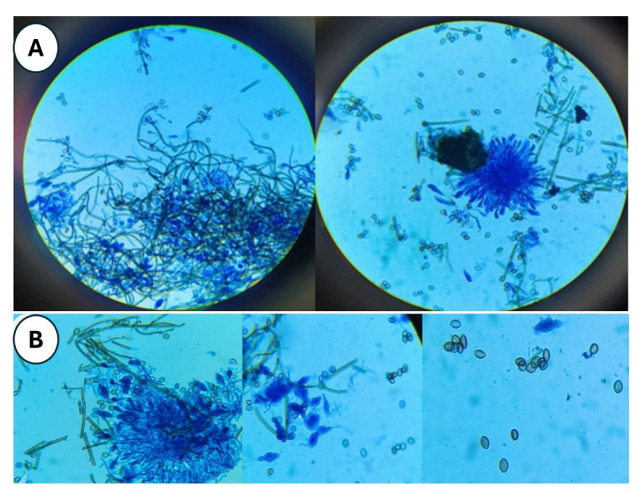
**(A)** Large, dark brown to black, flask-shaped perithecia with hair-like filamentous appendages (lactophenol cotton blue mount), **(B)** Close-up view of asci and single-celled ascospores: dark brown-colored septated hyphae and lemon-shaped ascospores (lactophenol cotton blue mount)

When fungal colonies were examined using slide cultures stained with lactophenol cotton blue, distinct morphological features were observed under light microscopy. The hyphae appeared brown and septate, while perithecia were large and dark brown to black, exhibiting a globose to flask-like shape with unbranched, hair-like filamentous appendages on their surface. These perithecia contained ostioles and enclosed asci, within which single-celled ascospores were present. The ascospores were olive brown in color and had a characteristic lemon-shaped appearance (Figures 2A and 2B).

Antifungal susceptibility testing was performed using the broth microdilution method in accordance with Clinical and Laboratory Standards Institute (CLSI) M38: *Reference Method for Broth Dilution Antifungal Susceptibility Testing of Filamentous Fungi* [ 3 ]. The testing was conducted in RPMI 1640 medium. Itraconazole exhibited the highest *in vitro* efficacy with a minimum inhibitory concentration (MIC) of 0.25 µg/mL, followed by terbinafine (8 µg/mL), griseofulvin (16 µg/mL), and fluconazole, which showed the least activity (MIC: 32 µg/mL). 

**Figure 3:**
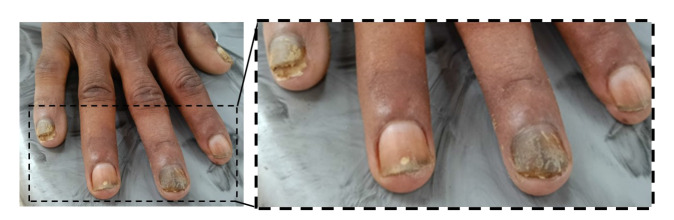
Follow-up photograph of the affected fingernail showing noticeable clinical improvement after treatment

The patient was treated with oral itraconazole (200 mg/day for 12 weeks) plus topical 5% amorolfine nail lacquer twice weekly, resulting in marked clinical improvement with reduced discoloration and subungual hyperkeratosis (Figure 3).

## Discussion

*Chaetomium globosum* is an uncommon cause of onychomycosis, primarily affecting immunocompromised individuals; however, cases in immunocompetent hosts have been reported. It is a saprophytic fungus commonly found in soil and decaying plant matter. *Chaetomium* is not commonly pathogenic; nevertheless, infections, such as cutaneous infections, onychomycosis, peritonitis, and brain abscesses have been reported [ 4 ]. The probable mode of infection in this case was traumatic inoculation while working in the fields.

Non-dermatophytic molds contribute to approximately 1.45-17.6% of cases [ 5 - 7 ]. Currently, the genus *Chaetomium* consists of 105 species since it was first described by Kunze in 1817. However, only a few species are recognized as human pathogens, including *C. atrobrunneum*, *C. strumarium*, *C. perlucidum*, *C. funicolum*, and *C. globosum*. Onychomycosis due to *C. globosum* is documented from India [ 8 , 9 ], Spain [ 10 ], Korea [ 1 ], Canada [ 11 , 12 ], and Japan [ 13 ].

The initial identification was based on morphological characteristics, revealing perithecia ranging from 175 to 280 µm in size, along with distinctive brown, septate setae. *Chaetomium globosum* was specifically identified by its brown, lemon-shaped ascospores measuring 9–12 × 7–9 µm and its inability to grow at a temperature of 42 °C. Clinical differentiation from dermatophytic onychomycosis is challenging, making mycological examination essential for accurate diagnosis [ 4 , 14 ]. MALDI-TOF provided reliable identification in the present case. However, sequence analysis of the internal transcribed spacer region of ribosomal DNA remains the gold standard for definitive species-level confirmation [ 15 ]. Nevertheless, it could not be performed due to resource limitations. This is acknowledged, and future studies should include this to improve the diagnosis of rare fungi.

Any disruption to the integrity of the corneal layer of the nail can allow fungi, including less pathogenic species, like *Chaetomium* spp., to penetrate more easily. Trauma is a key predisposing factor for onychomycosis and cutaneous lesions [ 16 ]. 

Additionally, occupation of an individual plays a crucial role in susceptibility to these infections. In the present case of nail infection, the patient was a mason, a profession that involves regular and prolonged exposure to materials, such as cement, sand, and soil. These substances are well-known reservoirs for various environmental fungi, including *C. globosum*. Additionally, the abrasive nature of cement may cause microabrasions to the nail bed and surrounding skin, allowing fungal entry and pathogenesis. Occupational exposure, along with trauma, likely contributed to the disruption of the corneal layer of the nail, facilitating fungal penetration.

In this case, antifungal testing showed itraconazole had the highest *in vitro* activity against *C. globosum* and was also effective clinically. These findings aligned with Guarro et al. [ 4 ], who found *Chaetomium* species susceptible to imidazoles, like itraconazole, while 5-fluorocytosine and amphotericin B were ineffective. Non-dermatophytic mold onychomycosis often responds poorly to standard antifungals [ 17 ]. Aspiroz et al. [ 10 ] achieved a complete cure with terbinafine; other studies support terbinafine and itraconazole efficacy. Similarly, in this case, oral itraconazole (200 mg/day) plus 5% amorolfine nail lacquer led to clinical improvement. However, complete clinical resolution could not be documented, as the patient did not return for extended follow-up after the initial improvement. The clinical outcome indicates that combining oral itraconazole with topical 5% amorolfine could be an effective treatment approach for rare non-dermatophytic fungi, like *Chaetomium globosum*.

## Conclusion

Onychomycosis is a frequently occurring nail infection, primarily caused by dermatophytes. In some instances, non-dermatophytic molds may also be involved, though they are often overlooked due to limited awareness. However, if clinical findings and laboratory results confirm a fungal etiology, these molds should be recognized as pathogens and treated appropriately with antifungal therapy.
